# Factors Associated With Successful In‐Office Diagnostic Vitreous Aspiration

**DOI:** 10.1155/joph/1407217

**Published:** 2026-11-14

**Authors:** Uyen Tu Nguyen, Joyce Kang, Hong Li, Kareem Moussa, Glenn Yiu

**Affiliations:** ^1^ Department of Ophthalmology and Vision Science University of California, Davis Sacramento USA ucdavis.edu; ^2^ School of Medicine University of California, Davis Sacramento USA ucdavis.edu; ^3^ Department of Public Health Sciences University of California, Davis Sacramento USA ucdavis.edu

**Keywords:** anterior paracentesis, diagnostic procedure, endophthalmitis, uveitis, vitrectomy, vitreous tap

## Abstract

**Background and Objective:**

To identify demographic, clinical, and procedural factors associated with successful vitreous taps and aspirate volume to optimize candidate selection and technique for this procedure.

**Study Design/Patients and Methods:**

We performed a retrospective analysis of patients who underwent in‐office diagnostic vitreous aspiration from January 2010 to December 2022. We reviewed patient demographics, clinical characteristics, and procedural factors and then performed univariate and multivariable regression analyses to identify factors associated with successful vitreous tap and aspirate volume.

**Results:**

Among 101 eyes from 101 patients (64% aged 60 years or older, 37.6% female) who received diagnostic vitreous taps in clinic, 77.2% were successfully performed, while the remainder required aqueous sampling. Regression analyses showed that past vitrectomy (*p* < 0.001) and smaller needle gauge (*p* = 0.001) were associated with successful taps, where eyes with prior vitrectomy had a 100% success rate as compared to 68% in eyes without prior vitrectomy. Interestingly, larger 25‐gauge needles were less successful (68%) than 27‐gauge needles (93%), likely due to documentation of only the largest needle gauge attempted. Eyes that underwent successful vitreous aspiration had a mean aspirate volume of 0.3 ± 0.1 mL, with higher volumes observed in phakic than pseudophakic eyes (0.3 ± 0.1 vs. 0.2 ± 0.1 mL, *p* = 0.034), as well as those with suspected or actual diagnoses of endogenous versus exogenous infectious uveitis (0.4 vs. 0.2 mL, *p* = 0.002 and 0.4 vs. 0.2 mL, *p* = 0.003, respectively).

**Conclusion:**

In‐office diagnostic vitreous taps are more likely to be successful in vitrectomized eyes but do not appear to be affected by other factors such as the patient’s age, severity or type of intraocular inflammation, or larger needle gauge.

**Summary**
• A retrospective study of 101 patients identified that prior vitrectomy and smaller needle gauge significantly improve the success rate of in‐office vitreous taps. Patients with prior vitrectomy achieved a 100% success rate, emphasizing the critical role of patient history and needle selection in optimizing vitreous aspiration procedures.

## 1. Introduction

Vitreous aspiration, also known as vitreous tap, serves as a valuable diagnostic and therapeutic tool for various ocular conditions, including intraocular infections, inflammatory conditions, and malignancies [1]. This minimally invasive procedure provides a direct sample of vitreous humor, allowing for precise identification of causative agents and selection of targeted therapies. Moreover, vitreous needle aspiration can be performed both inpatient and in‐clinic, making it an easily accessible diagnostic tool for urgent cases. However, despite its simplicity and safety, the procedure can be painful. A failed tap that does not yield an adequate sample may require repeat needle insertions or subsequent anterior paracentesis, leading to further patient distress [2].

Previous studies have helped optimize vitreous aspiration techniques, but demographic, clinical, and procedural factors associated with a successful in‐clinic tap have not been reported [3, 4]. Success in vitreous aspiration is typically defined by obtaining an adequate sample volume, typically ranging from 0.1 to 0.3 mL, for diagnostic testing [5]. The procedure involves using a needle ranging from 22‐gauge to 30‐gauge, connected to a syringe and inserted through the pars plana to aspirate 0.2‐0.3 cc of vitreous [6]. An unsuccessful attempt, known as a dry tap, occurs when no adequate sample is obtained, which may necessitate repeat attempts or alternative sampling methods [7]. In this study, we performed a retrospective analysis of vitreous aspiration performed for a variety of clinical indications to identify factors that contribute to successful vitreous aspirations, with the goal of enhancing candidate selection and refining the technique.

## 2. Patients/Materials and Methods

### 2.1. Patient Selection

We performed a retrospective review of patients who underwent diagnostic in‐office vitreous aspiration (CPT 67015) or anterior paracentesis (CPT 65800) due to suspected endophthalmitis or other forms of uveitis at the University of California, Davis Eye Center. Approval was obtained from our Institutional Review Board to conduct a search of our electronic health records from January 2010 to December 2022, and the study adhered to the tenets of the Declaration of Helsinki. Only procedures associated with a valid procedure note and informed consent obtained from patients were included. Patients who received aqueous sampling without documentation of previously attempted vitreous taps were excluded from our study.

### 2.2. Clinical Review

We reviewed clinical records to collect demographic information including age and sex, ocular characteristics such as eye laterality, lens status, and history of past vitrectomy, as well as clinical details such as presence of hypopyon, presence of vitreous haze, suspected diagnosis, final diagnosis, and culture results. We also reviewed procedure notes to document procedural factors such as needle gauge (25‐gauge, 27‐gauge, or 30‐gauge), quadrant location (superotemporal or inferotemporal), type of anesthesia (topical only, subconjunctival, or peribulbar block), and volume of vitreous aspirate. In eyes where the vitreous tap was unsuccessful and an anterior chamber paracentesis was performed, we collected details of the aqueous tap including needle gauge and volume of aqueous aspirate.

### 2.3. Statistical Analysis

We performed univariate logistic regressions to identify factors associated with successful vitreous tap. In eyes that had successful vitreous taps, we performed univariate linear regressions to identify factors associated with the vitreous aspirate volume. We included variables with *p* < 0.20 in a multivariable linear regression model, with vitreous aspirate volume as the dependent variable. Because suspected diagnosis and actual diagnosis had related diagnostic classifications, we did not include both variables in the same multivariable model. Actual diagnosis was selected for the final model because it represented the final diagnostic classification. We evaluated multivariable models using aspirate volume on its original and natural‐log scale and assessed residual normality through visual inspection of residual histograms.

Patient age was categorized as greater than 60 or 60 years and younger, based on the mean age of posterior vitreous detachment onset [4]. All hypothesis tests were two sided and evaluated at a significance level of *p* < 0.05. Variables that are statistically significant on univariate regression were analyzed in a multivariable model. Statistical analyses were performed using SPSS Statistics (Version 22; IBM, Armonk, NY, USA).

## 3. Results

### 3.1. Clinical and Procedural Characteristics

We identified 101 eyes from 101 patients (64.4% aged 60 years or older, 62.4% female) who underwent diagnostic in‐office vitreous aspiration. The proportion of left and right eyes were similar (47.5% vs. 52.5%, respectively), with 54 phakic (53.4%), 42 pseudophakic (41.6%), and 5 aphakic (5%) eyes. Based on medical records, 25.7% had prior vitrectomy surgery. At presentation, 40.6% had a hypopyon and 21.8% had vitreous haze documented. Suspected diagnoses at the time of the vitreous tap included 30 cases of endogenous infectious uveitis, 62 exogenous infectious uveitis, and 9 noninfectious uveitis. The final diagnoses based on clinical course and culture results included 26 endogenous infectious uveitis, 61 exogenous infectious uveitis, and 14 noninfectious uveitis, with 22.8% of the cases showing positive cultures where a pathologic organism was isolated (Table 1).

**TABLE 1:** Factors associated with successful in‐office vitreous taps.

	All patients	Successful tap	Failed tap	Univariate regression	
	*n* = 101	*n* = 78	*n* = 23	Odds ratio	*p* value
Age					
60+	65 (64.4%)	51 (78.5%)	14 (21.5%)	Reference	0.69
< 60	36 (35.6%)	27 (75.0%)	9 (25.0%)	0.8	
Sex					
Female	63 (62.4%)	49 (77.8%)	14 (22.2%)	Reference	0.87
Male	38 (37.6%)	29 (76.3%)	9 (23.7%)	0.9	
Laterality					
Right eye	53 (52.5%)	38 (71.7%)	15 (28.3%)	Reference	0.16
Left eye	48 (47.5%)	40 (83.3%)	8 (16.7%)	2.0	
Past vitrectomy					
No	75 (74.3%)	52 (69.3%)	23 (30.7%)	Reference	0.001^∗^
Yes	26 (25.7%)	26 (100%)	0 (0%)	∞	
Lens status					
Phakic	54 (53.4%)	43 (79.6%)	11 (20.4%)	Reference	0.29
Pseudophakic	42 (41.6%)	30 (71.4%)	12 (28.6%)	0.6	
Aphakic	5 (5%)	5 (100%)	0 (0%)	—	
Hypopyon presence					
No	60 (59.4%)	43 (71.7%)	17 (28.2%)	Reference	0.30
Yes	41 (40.6%)	35 (85.4%)	6 (14.6%)	2.3	
Vitreous haze					
No	79 (78.2%)	61 (77.2%)	18 (22.8%)	Reference	0.99
Yes	22 (21.8%)	17 (77.3%)	5 (22.7%)	1.0	
Suspected diagnosis					
Exogenous infection uveitis	62 (61.4%)	49 (79.0%)	13 (21.0%)	Reference	0.10
Endogenous infectious uveitis	30 (29.7%)	20 (66.7%)	10 (33.3%)	0.5	
Noninfectious uveitis	9 (8.9%)	9 (100%)	0 (0%)	—	
Actual Diagnosis					
Exogenous infection uveitis	61 (60.4%)	49 (80.3%)	12 (19.7%)	Reference	0.23
Endogenous infectious uveitis	26 (25.7%)	17 (65.4%)	9 (34.6%)	0.5	
Noninfectious uveitis	14 (13.9%)	12 (85.7%)	2 (14.3%)	3.1	
Culture					
Negative	78 (77.2%)	57 (73.1%)	21 (26.9%)	Reference	0.07
Positive	23 (22.8%)	21 (91.3%)	2 (8.7%)	0.26	
Needle gauge					
23 or 25	48 (47.5%)	32 (66.7%)	16 (33.3%)	Reference	0.002^∗^
27 or 30	50 (49.5%)	46 (92.0%)	4 (8.0%)	5.8	
Anesthesia					
Subconjunctival lidocaine	94 (93%)	74 (78.7%)	20 (21.3%)	Reference	0.21
Topical anesthetics	5 (5%)	3 (60.0%)	2 (40.0%)	0.4	
Peribulbar block	2 (2%)	1 (50.0%)	1 (50.0%)	0.3	
Quadrant of injection					
Supratemporal	58 (57.4%)	44 (75.9%)	14 (24.1%)	Reference	> 0.99
Infratemporal	29 (28.7)	22 (75.9%)	7 (24.1%)	1.0	

^∗^ *p* value < 0.05.

Most vitreous taps were performed using 25‐gauge (*n* = 47) or 27‐gauge (*n* = 45) needles, while 30‐gauge needles were only used for 5 eyes with a history of past vitrectomies. Most used subconjunctival anesthetic (*n* = 94), whereas few taps were performed with topical anesthetic only (*n* = 5) or peribulbar block (*n* = 2). The majority of vitreous taps were performed in the superotemporal (58 eyes) versus inferotemporal quadrant (29 eyes).

### 3.2. Factors Associated With Successful Vitreous Taps

The overall success rate for obtaining a vitreous specimen with in‐office vitreous taps was 77.2%, while the remainder required aqueous sampling. On univariate regression analyses, eyes that had past vitrectomy were strongly associated with successful vitreous aspiration (OR ∞, *p* < 0.001), as all 26 vitrectomized eyes had successful taps. In contrast, eyes without past vitrectomy had a 68% success rate. In a subanalysis limited to eyes without prior vitrectomy, the use of a smaller 27‐gauge needle compared to a larger 25‐gauge needle was associated with a higher likelihood of success (*p* = 0.03), with success rates of 87% for 27‐gauge and 63% for 25‐gauge needles. The use of 30‐gauge needles was excluded from this analysis because it was exclusively used in vitrectomized eyes.

### 3.3. Factors Associated With Vitreous Aspirate Volume

Among eyes that successfully underwent vitreous aspiration, the mean volume of the vitreous aspirate was 0.3 ± 0.1 mL. Phakic eyes had higher mean aspirate volume than pseudophakic eyes (0.3 ± 0.1 vs. 0.2 ± 0.1 mL; *p* = 0.034). Additionally, eyes with suspected endogenous infectious uveitis had higher aspirate volumes than those with suspected exogenous infectious uveitis (0.4 vs. 0.2 mL; *p* = 0.002). Similarly, eyes with a final diagnosis of endogenous infectious uveitis had greater aspirate volumes than those with a final diagnosis of exogenous infectious uveitis (0.4 vs. 0.2 mL; *p* = 0.003). Both aspirate volume and residuals from the original‐scale multivariable model were right‐skewed. After log‐transformation of aspirate volume, residuals more closely approximated a normal distribution (Figure 1A–C). In this model, neither lens status (*p* = 0.35) nor final diagnosis (*p* = 0.30) showed significant association with aspirate volume (Table 2).

**FIGURE 1:**
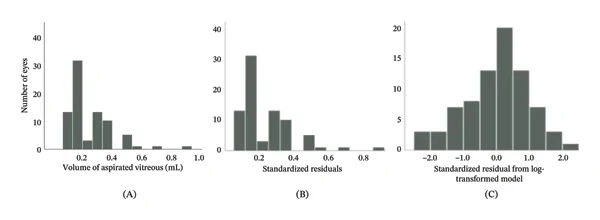
Histograms of (A) aspirated vitreous volume, (B) residuals from multivariable linear model using original scale, and (C) residuals from multivariable linear model using log‐transformed scale.

**TABLE 2:** Factors associated with aspirated vitreous volume.

	All patients *n* = 78	Mean volume aspirated (mL) ± SD	Univariate regression *p* value	Adjusted *β* (95% CI), log scale	Multivariable regression *p* value
Age					
60+	51 (65.4%)	0.3 ± 0.2	0. 93		—
< 60	27 (34.6%)	0.3 ± 0.1			
Sex					
Female	29 (37.2%)	0.2 ± 0.1	0.37		—
Male	49 (62.8%)	0.3 ± 0.2			
Past vitrectomy					
Yes	26 (33.3%)	0.2 ± 0.1	0.39		—
No	52 (66.7%)	0.3 ± 0.2			
Lens status					0.35
Phakic	43 (55.1%)	0.3 ± 0.2	0.03^∗^	Reference	Reference
Pseudophakic	30 (38.5%)	0.2 ± 0.1		−0.05 (−0.54–0.44)	0.36
Aphakic	5 (6.4%)	0.3 ± 0.1		−0.23 (−0.71–0.26)	0.84
Hypopyon presence					
Yes	35 (44.9%)	0.3 ± 0.1	0.56		—
No	43 (55.1%)	0.3 ± 0.2			
Vitreous haze					
Yes	17 (21.8%)	0.3 ± 0.2	0.56		—
No	61 (78.2%)	0.3 ± 0.1			
Suspected diagnosis					
Endogenous infection uveitis	20 (25.6%)	0.3 ± 0.2	0.002^∗^		—
Exogenous infectious uveitis	49 (62.8%)	0.2 ± 0.1			
Noninfectious uveitis	9 (11.5%)	0.3 ± 0.1			
Actual diagnosis					0.30
Endogenous infection uveitis	17 (21.8%)	0.4 ± 0.2	0.003^∗^	−0.24 (−0.56–0.08)	0.13
Exogenous infectious uveitis	49 (62.8%)	0.2 ± 0.1		Reference	Reference
Noninfectious uveitis	12 (15.4%)	0.3 ± 0.1		−0.12 (−0.51–0.27)	0.54
Needle gauge					
25	32 (41.0%)	0.3 ± 0.2	0.34		—
27 or 30	46 (59.0%)	0.2 ± 0.1			
Quadrant of injection					—
Inferotemporal	22 (28.2%)	0.2 ± 0.1	0.91		
Supratemporal	44 (56.4%)	0.3 ± 0.2			
Unknown	12 (15.4%)	0.3 ± 0.1			

*Note:* The adjusted model uses natural‐log‐transformed aspirate volume.

^∗^ *p* value < 0.05.

## 4. Discussion

Vitreous aspiration enables prompt diagnosis and guides treatment decisions in eyes with ocular inflammation. While in‐office taps are a common method of vitreous sampling [2, 8–11], the gel‐like quality of vitreous fluid can pose a challenge, sometimes requiring multiple attempts or substitution with aqueous sampling that can lead to pain and distress for patients. Here, we explored demographic, clinical, and procedural factors associated with successful in‐clinic vitreous taps to optimize the technique and stratify appropriate candidates for the procedure.

Despite vitreous liquefaction occurring with age [12], we did not observe a relationship between age and successful taps. Specifically, 64.4% of the patients were 60 years and older, while 35.6% were younger than 60, with no statistically significant difference in success rates between these age groups (*p* = 0.928). Instead, eyes that had past vitrectomy had a 100% success rate, which was expected since the vitreous cavity has been replaced with aqueous fluid that is much easier to aspirate [13]. Interestingly, the use of a smaller 27‐gauge needle had a higher success rate than 25‐gauge needles (87% vs. 63%) among eyes without prior vitrectomy (*p* = 0.03). This finding is, however, confounded by the fact that smaller gauge needles were exclusively used in vitrectomized eyes, making it difficult to determine if the association with needle gauge was an independent factor. Multivariable regression could not be performed because 30‐gauge needles were exclusively used in eyes with past vitrectomy, and all vitrectomized eyes had successful vitreous taps. Future studies using a larger and more heterogeneous patient cohort could provide greater sensitivity for detecting clinical or procedural factors associated with in‐office vitreous tap success.

While our study does not support the use of larger needle gauge to increase the chance of successful vitreous aspirations, prior studies suggest that smaller needles may also increase diagnostic yield. Vitreous sampling using 30‐gauge needles yielded 80% positive microbiological cultures as compared to 31% from 25‐ and 27‐gauge needles (*p* < 0.01) [3]. Another study showed that the vacuum force generated by syringes may more dependent on syringe volume than needle gauge [14]. Thus, a smaller gauge needle on a larger syringe could provide greater vacuum force and provide greater diagnostic yield, without sacrificing patient comfort. Needle diameters impact vacuum generation over time, so a slower speed of aspiration may be needed for optimal vitreous aspiration.

Our study found that greater aspirate volumes were collected from eyes that were phakic, had prior vitrectomy, or had a suspected or final diagnosis of endogenous versus exogenous infectious uveitis. It is important to note, however, that these differences reflect how much volume was drawn, rather than how much could be drawn. Nevertheless, we hypothesize that prior vitrectomy improves the ease of vitreous aspiration, while the rationale for the difference based on lens status is unclear. Further research could provide insight on whether vitreous viscosity may differ between different etiologies which can impact the ease and extent of fluid aspiration.

Our study is limited by its retrospective design and limited sample size. Clinical documentation provides only information on the final procedure performed and rarely provides insight on operator intent, procedural effort, or other unsuccessful attempts. For example, a physician who attempted different needles may only document the largest needle gauge attempted. Some procedural details were not consistently recorded, and clinician preference may vary between individuals. Syringe sizes, for example, were rarely documented. In the absence of a standardized protocol, clinicians selected syringe volumes based on preference. This variability and limited documentation restricted our ability to evaluate how syringe volume may influence the ease of vitreous aspiration. Nevertheless, our study confirms that vitreous taps are easier to perform in vitrectomized eyes, while most demographic, clinical, or procedural factors such as age, severity of inflammation, and needle gauge do not appear to impact likelihood of success. These insights can guide clinicians in patient selection and techniques for in‐office vitreous aspiration to improve diagnostic yield and patient outcomes.

## Funding

No funding was received for this manuscript.

## Disclosure

The funding organizations did not play any role in the design or conduct of this retrospective study.

## Conflicts of Interest

Glenn Yiu received consulting fees from 4DMT, Abbvie, Adverum, Alcon, Bausch & Lomb, Boehringer Ingelheim, Clearside Biomedical, Endogena Therapeutics, Genentech, Iridex, Janssen, jCyte, Myrobalan Therapeutics, NGM Biopharmaceutical, Novartis, Ocuphire, Opthea, Ray BioTherapeutics, RegenXBio, Samsung Bioepis, Stealth Therapeutics, Supra Medical, Surrozen, and West Pharmaceutical. The other authors declare no conflicts of interest.

## Data Availability

The data that support the findings of this study are available on request from the corresponding author. The data are not publicly available due to privacy or ethical restrictions.
